# Description of the “Brigade Case”

**Published:** 1861-09

**Authors:** H. S. Hewit

**Affiliations:** Formerly Assistant Surgeon U. S. A.; and Respectfully Submitted for the Approval of the Medical Staff of the U. S. Army


### DESCRIPTION OF THE "BRIGADE CASE,"

DESIGNED BY

H. S. HEWIT, M. D.,

*Formerly Assistant Surgeon U. S. A.*

AND RESPECTFULLY SUBMITTED FOR THE APPROVAL OF THE MEDICAL STAFF OF THE U. S. ARMY.

---

The case described below is one designed by the writer to meet a surgical want hitherto unsatisfied. It is intended to contain every instrument which can be useful in any emergency, and, with the instruments already in possession of the staff, will furnish a complete *armamentarium chirurgicum.*

It consists of the following instruments:

*For Amputations.*—Four amputating knives; two amputating scalpels; one amputating tenaculum; one capital saw; one finger saw; two spiral tourniqu[illegible]

*For Trephining.*—Two trephine[illegible]e Hey's saw; one elevator.

*Forceps.*—One Liston's straight bone forceps; one Isaac's bayonet do.; two Lüer's bone-gnawing forceps; one Strohmeyer's stumpholding forceps; two tooth forceps; two Lüer's artery forceps; one torsion forceps; one thumb forceps; one mouse-tooth forceps.

*Saws.*—One Strohmeyer's saw; one saw à dos mobile; one saw guard.

*Trocars and Catheters.*—One curved rectum trocar; one straight trocar; one partition catheter; five silver catheters, 1, 3, 5, 7, 9; one silver catheter for prostrate, 12; one steel staff grooved; twelve English flexible catheters.

*Needles.*—One Mott's artery needle; one right Deschamps' artery needle; one left Deschamps' artery needle.

*Bistouries and Scalpels, &c., &c.*—One sharp-pointed straight bistoury; one probe-pointed straight bistoury; one probe-pointed curved bistoury; one sharp-pointed curved bistoury; one hernia bistoury; four scalpels; one tenaculum; two double hooks, sharp; two double hooks, blunt; two retractors; one pair of Musseux's forceps; one pair of polypus forceps; one pair of dressing forceps; one pair of heavy straight scissors; one pair of ordinary straight scissors; one pair of curved scissors; one silver director; one steel director; one Schleswig bullet forceps; one Hamilton's bullet forceps; two double trachea tubes; one Lüer's articulated œsophagus tube; one wire suture needle; two eye needles; one vaccinating scarificator; one hard rubber four-ounce syringe; silver probes, wire, and suture silk.

It will be observed that amputating, trephining, resecting, and artery instruments are here comprised, together with tracheotomy tubes, trocars, and the silver suture needle.

The dimensions of the case are, length, 18 inches; breadth, $13\frac{3}{4}$ inches; depth, $2\frac{1}{2}$ inches. The weight is $19\frac{3}{4}$ pounds, and with the containing leather valise, like case, will be upwards of thirty pounds.

It is intended that the exterior case shall be made five inches in depth, the lid to contain rollers two and a half inches wide by seven yards long, placed on end, and lint, cerate, oil, chloroform, and sponges, so that with this case occupying no more room than a common travelling valise, any amputation, resection, ligature, or other operation can be performed, or any wo[illegible] dressed except those requiring spints.

The undersigne[illegible]ectfully recommends that one case similar to the abo[illegible] furnished to every surgeon and medical director of [illegible]regular army, and to every brigade surgeon of the volunteer forces.

The case itself is made of tin, japanned, and is as compact and light as a complete case can be made.

It is not intended for hand transportation, excepting for short distances. It is of convenient size for any other conveyance or for packing.

The instruments have been made and arranged by Mr. Jules Teincken, of Astor Place, agent for A. Lüer, of Paris, whose name alone is a guarantee of excellence of material and workmanship.

The present aspect of surgery, and the experience of the Schleswig-Holstein war and the Crimean war, urge imperatively upon all military surgeons the cultivation, according to their means and circumstances, of conservative surgery. The above case furnishes all the instruments necessary for resecting bones or joints, excepting the chain saw, which has been omitted on account of its extreme liability to get out of order. The saws in the case, it is believed, will answer every purpose of the chain saw.

The long, hollow, silver suture needle is added under the belief that silver or other metallic sutures are destined to occupy a high place in both ordinary and conservative surgery.

It is the intention of the writer to make trial of the silver thread if he has opportunity (silver wire twisted over silk) both as ligatures and sutures, and report the result.

The high claims of the profession, the future of medical and surgical science, and the great and ever present cause of humanity urge the profession, both military and civil, to high heroic and noble enterprise.

Let us see that history record that no life or limb was sacrificed in the present war which sanitary science and foresight or surgical skill could have saved; and let our profession seize the present glorious opportunity to demonstrate its value and utility in times of real danger and distress. If quackery hereafter has a front to show, it will be simply our own fault. —*Am. Med. Times.*

